# Aircraft and Risk of Importing a New Vector of Visceral Leishmaniasis

**DOI:** 10.3201/eid1707.102002

**Published:** 2011-07

**Authors:** Carlos H.N. Costa, Isabel K.F. de Miranda-Santos

**Affiliations:** Author affiliations: Federal University of Piauí, Brazil (C.H.N. Costa); University of São Paulo at Ribeirão Preto, Brazil (I.K.F. de Miranda-Santos)

**Keywords:** vector-borne infections, sand fly, leishmaniasis, kala-azar, Leishmania infantum, Lutzomyia longipalpis, travel, Brazil, letter

**To the Editor**: Kala-azar, or visceral leishmaniasis, is a parasitic disease that leads to fever, anemia, and hepatosplenomegaly. Death is the usual outcome when infection is not treated. The majority of infections are caused by the protozoan *Leishmania donovani*, restricted to India and eastern Africa, but the most widespread are caused by *L. infantum*, found from People’s Republic of China to the New World, where it infects humans, dogs, and wild canids. All Mediterranean countries are affected by *L. infantum*, where most patients are co-infected with HIV. Several species of sand flies transmit the disease (*1*).

During the 1980s, urban transmission of kala-azar became a major problem in Brazil. More than 3,000 cases are reported annually, and the disease has spread from northeastern Brazil westward to the Amazon region, as well as to the industrialized southeast. Several as yet unproven explanations for the urbanization of kala-azar in Brazil have been proposed (*2*), but whatever the reason, it is associated with proliferation of *Lutzomyia longipalpis* sand flies, which, in turn, are strongly associated with human environments. The vector can easily spread by entering buses or trains looking for food at night or for hiding places at dawn. Invasion of new areas by sand flies through transportation of ornamental plants has been observed (R. Brazil, pers. comm.), possibly by insect eggs or larvae being carried in organic matter.

Kala-azar has now reached the temperate Brazilian south and Argentina. This spread of the disease warns us of the danger of introduction in other temperate areas. Europe is particularly vulnerable because of the existing natural transmission of *L. infantum*. This risk is increased by recently created daily direct flights to Lisbon from Fortaleza, Natal, Brasília, and Belo Horizonte (Figure), Brazilian cities where epidemics of the disease have occurred. Lisbon is suitable to canine infection, and >10% of dogs may be infected (*3*). The climate is a barrier for the introduction of many vectors outside their normal range, such as *Anopheles gambiae* mosquitoes in temperate zones (*4**,**5*), but the threshold of change for *L. longipalpis* sand flies is minimal. The Mediterranean area is as dry as northeastern Brazil, where the disease is now highly endemic. Furthermore, the annual average temperature and cooler months in Lisbon (at 38°44′N) are only 3–4°C lower than those of São Borja, Rio Grande do Sul state, the southernmost city where *L. longipalpis* transmits kala-azar, and even warmer than Chajarí, Argentina (at 30°46′S, ≈500 km from Buenos Aires and only 8° farther from a pole than Lisbon), at the highest southern latitude where this vector is found (*6*).

**Figure:**
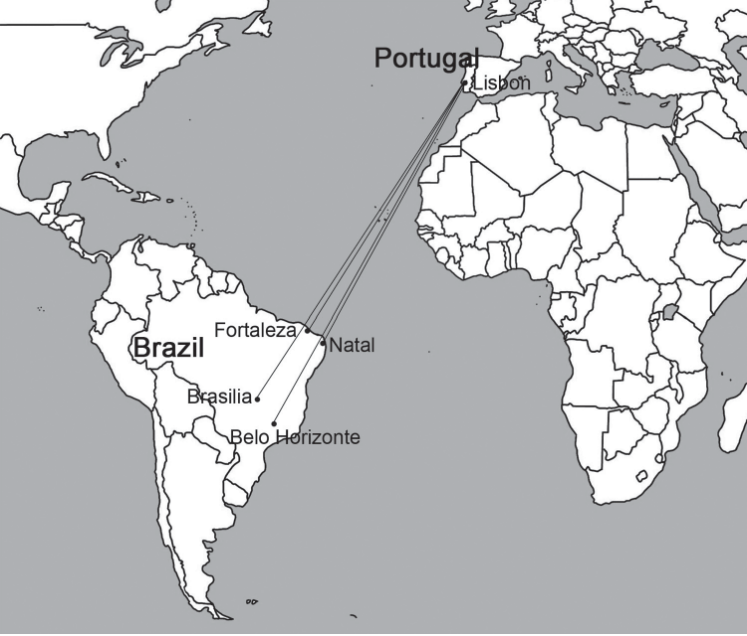
Commercial air transport routes between Lisbon, Portugal, and cities in Brazil that could make possible the accidental importation into Europe of *Lutzomyia longipalpis* sand flies, a vector of visceral leishmaniasis.

Human kala-azar is less common in Europe, possibly because sand flies there are less anthropophilic. If aircraft introduce anthropophilic *L. longipalpis* sand flies in Lisbon, the situation could change dramatically, and kala-azar might become a major urban disease in Europe. The International Health Regulations recommends disinfection of aircraft by preflight and blocks-away spraying with pyrethroids (*7*). However, significantly reduced susceptibility to pyrethroids in wild populations of *L. longipalpis* sand flies was recently described in Brazil (*8*). Centuries after its introduction to South America by Iberian colonizers, kala-azar may make its way back to Europe with a more forceful vector—this time by air, not by sea. To reduce this risk, much information needs to be known about the biology of *L. longipalpis* sand flies, such as minimum temperature tolerance, mechanisms of urban spread, presence in aircraft, and role in inducing more severe disease._______________________________________________________________________________________________________________________________
